# Individual metabolic brain network abnormalities associated with drug-resistant mTLE vary in surgical outcomes

**DOI:** 10.3389/fneur.2024.1444787

**Published:** 2024-12-18

**Authors:** Xinyi Wang, Pan Zhang, Dandan Lin, Chunlei Zhao, Zhifeng Huang, Ziqian Chen, Hui Li, Shangwen Xu

**Affiliations:** ^1^Department of Radiology, Shengli Clinical Medical College of Fujian Medical University, Fujian Provincial Hospital, Fuzhou University Affiliated Provincial Hospital, Fuzhou, China; ^2^Department of Diagnostic Radiology, Fuzong Clinical Medical College of Fujian Medical University, Fuzhou, Fujian, China; ^3^Department of Diagnostic Radiology, The 900th Hospital of Joint Logistic Support Force, PLA, Fuzhou, Fujian, China; ^4^Medicine Department, Fujian Health College, Fuzhou, Fujian, China

**Keywords:** drug-resistant mesial temporal lobe epilepsy, positron emission tomography-computed tomography, connectivity analysis, effect size, surgical outcomes

## Abstract

**Objective:**

This investigation aimed to elucidate alterations in metabolic brain network connectivity in drug-resistant mesial temporal lobe epilepsy (DR-MTLE) patients, relating these changes to varying surgical outcomes.

**Methods:**

A retrospective cohort of 87 DR-MTLE patients who underwent selective amygdalohippocampectomy was analyzed. Patients were categorized based on Engel surgical outcome classification into seizure-free (SF) or non-seizure-free (NSF) groups. Additionally, 38 healthy individuals constituted a control group (HC). Employing effect size (ES) methodology, we constructed individualized metabolic brain networks and compared metabolic connectivity matrices across these groups using the DPABINet toolbox.

**Results:**

Compared to HCs, both SF and NSF groups exhibited diminished metabolic connectivity, with the NSF group showing pronounced reductions across the whole brain. Notably, the NSF group demonstrated weaker metabolic links between key networks, including the default mode network (DMN), frontoparietal network (FPN), and visual network (VN), in comparison to the SF group.

**Conclusion:**

Individual metabolic brain networks, constructed via ES methodology, revealed significant disruptions in DR-MTLE patients, predominantly in the NSF group. These alterations, particularly between limbic structures and cognitive networks like the DMN, suggested impaired and inefficient information processing across the brain’s networks. This study identified abnormal brain networks associated with DR-MTLE and, importantly, contributed novel insights into the mechanisms underlying poor postoperative seizure control, and offered potential implications for refining preoperative assessments.

## 1 Introduction

Drug-resistant mesial temporal lobe epilepsy (DR-MTLE), a predominant form of intractable epilepsy in clinical practice, originates in the medial structures of the temporal lobe, including the hippocampus, parahippocampal gyrus, and amygdala. Surgical resection, specifically selective amygdalohippocampectomy, has emerged as an effective intervention for DR-MTLE. However, the persistence of seizures postoperatively in approximately 30% of patients underscores the complexity of this neurological disorder, which affects multiple brain regions beyond the epileptogenic focus (1–5).

The comprehensive pre-surgical assessment of DR-MTLE, integrating clinical, electrophysiological, neuropsychiatric, and imaging evaluations, is crucial yet challenging due to the uncertain predictive ability of these assessments for postoperative seizure outcomes. Within this context, ^18^F-FDG PET imaging, a neuroimaging technique that measures brain glucose metabolism, can detect neuronal function more directly and quantitatively than BOLD-fMRI. It reveals brain activity by linking synaptic activity with local energy consumption and is characterized by high reproducibility and a favorable signal-to-noise ratio (6, 7).

Previous studies (8–12) employing ^18^F-FDG PET have identified abnormal metabolic network connections in DR-MTLE patients, exploring these connections’ influence on postoperative seizure control. However, these studies (10, 13, 14) often adopt a group-level approach, potentially overlooking individual-specific network information (15). It is thus that debate is ongoing on best practices for calculating and validating metabolic connectivity, and individual-level metabolic connectivity in particular (16). Recent advancements in effect size (ES) methodology (17) and distribution dispersion (15, 18, 19) have enabled the construction of individual metabolic brain networks, offering new avenues for studying epileptic brain networks (20). In addition, unlike these studies based on static FDG data, some studies (21, 22) have used the time series of single-subject dynamic FDG data from healthy populations to calculate individual-level metabolic connectivity.

This study involved a retrospective analysis of DR-MTLE patients who underwent standardized surgical resection, with a focus on individual-level brain metabolic connections and their association with postoperative seizure recurrence. By leveraging the ES methodology, the study aims to delve into the metabolic mechanisms behind varying surgical outcomes, offering a new perspective on the interplay between epilepsy and brain metabolic networks.

## 2 Materials and methods

### 2.1 Study participants

This study retrospectively analyzed 87 right-handed patients diagnosed with drug-resistant mesial temporal lobe epilepsy (DR-MTLE) and undergoing ^18^F-FDG-PET imaging between January 2011 and October 2020, who underwent selective amygdalohippocampectomy at our center. These patients were followed up for at least one year. Additionally, 38 healthy individuals formed the control group (HC). Participants were selected based on comprehensive evaluations including clinical history, physical examination, EEG records, preoperative MRI, ^18^F-FDG PET scanning and postoperative pathological results, adhering to the diagnostic criteria of the International League Against Epilepsy. Exclusion criteria included the followings: (a) Younger than 16 years old (younger children have immature brain tissue development); (b) Lateral temporal lobe epilepsy or extra-temporal lobe epilepsy; (c) Obvious structural lesions on MRI such as ischemia, hemorrhage, infarction and tumor; (d) Severe systemic diseases such as diabetes, liver and kidney failure, cardiovascular and cerebrovascular diseases; (e) Clinical onset or EEG detection of epileptiform discharges within 24 h prior to PET examination; (f) non-cooperation during examinations. After screening, a total of 87 patients were included in the study. Patients were then classified by the Engel surgical outcome scale (23) as seizure-free (SF: Engel Ia) and non-seizure-free (NSF: Engel Ib-IV). This study was ethically approved, and informed consent was obtained from all participants.

### 2.2 ^18^F-FDG PET imaging protocol

Participants underwent ^18^F-FDG PET imaging, following a strict protocol that included a 12-h fast and abstinence from drugs influencing glucose metabolism. The PET scans were performed using a General Discovery LS PET scanner, 50 min post-intravenous injection of 0.15 mCi/kg FDG. Scans were acquired over 15 min and reconstructed on a 128 × 128 × 35 matrix in Analyze format.

### 2.3 PET data preprocessing

Data preprocessing utilized Statistical Parametric Mapping12 (SPM12) (24). The steps included spatial normalization to the Montreal Neurological Institute brain space using Chinese-specific PET templates (25). Finally, a Gaussian kernel with a full width of 16 mm at half maximum was applied to smooth the standardized PET images. Voxel intensities were normalized to the mean brain intensity for each participant.

### 2.4 Construction of individual metabolic brain networks

Individual metabolic brain networks were constructed using the automated anatomical labeling (AAL) atlas (26) to define 116 regions of interest (ROIs), each associated with a corresponding resting-state brain network (27). The standardized uptake value ratio (SUVR) relative to cerebellum was calculated for each ROI (28). Correlation coefficients between ROIs were computed to create weighted undirected network matrices (17, 29, 30), applying a threshold to determine the presence of connections. To reduce the complexity of connectivity network visualization and to control Type I error in statistics, this study set the threshold at 0.017 (0.05/3). The ES methodology (17) applied to individual ^18^F-FDG-PET image for constructing a correlation matrix which included pairwise regional metabolic connections. It utilizes the ES difference of regional SUVR between individual subjects and the HC group to generate an individual metabolic network. Detailed formulas included in this method can be found in the referenced article. The summary of all computational steps is as follows:

1. All PET images were spatially normalized, and the SUVR values were calculated.
2. A correlation coefficient matrix for the HC group was created using traditional group-level methods.
3. The mean and standard deviation of SUVR values between pairs of brain regions in the HC group were calculated.
4. The effect size difference between any two brain regions was computed.
5. The Fisher transformation was applied to obtain the correlation coefficient R.
6. The weighting matrix was calculated as W = 1-R.
7. The calculation of the individual correlation coefficient matrix, which is the product of the weight matrix and the group-level metabolic brain network matrix of the HC group.

### 2.5 Statistical analysis

Comparative analyses between seizure-free (SF), non-seizure-free (NSF), and HC groups were conducted using independent two-sample t-tests, one-way ANOVA, and chi-square tests. The DPABIINet toolbox facilitated the analysis of metabolic connectivity, the inter-group differences among the SF group, NSF group, and HC group were investigated using analysis of covariance (ANCOVA) with age as a covariate, and the distribution of inter-group differences was obtained through 5,000 permutation tests. The significance level was set at *p* < 0.05 and multiple comparisons were corrected using the false discovery rate (FDR). In the presence of significant ANCOVA results, pairwise statistical tests were conducted between the three groups using independent samples *t*-tests, with intergroup differences in metabolic connectivity evaluated via 5,000 permutation tests, and a significance threshold of *p* < 0.017, followed by FDR correction for multiple comparisons.

## 3 Results

### 3.1 Demographic and clinical characteristics

Our study categorized 87 DR-MTLE patients postoperatively into seizure-free (SF, *n* = 37) and non-seizure-free (NSF, *n* = 50) groups, according to the Engel surgical outcome classification. Age differences between the SF, NSF, and healthy control (HC) groups were statistically significant (*p* < 0.05), while gender distribution was comparable (*p* > 0.05). No significant differences were observed between SF and NSF groups in terms of clinical features, including seizure history, disease duration, and presence of hippocampal atrophy (*p* > 0.05) (refer to Table 1 the for comprehensive demographic and clinical data).

**Table 1:** General clinical data of the SF, NSF, and HC groups.

	SF (*n* = 37)	NSF (*n* = 50)	HC (*n* = 38)	*p* value
Age (year)	26.59 ± 6.47	29.36 ± 6.77	36.0 ± 1.7	0.000*
Gender (male/female)	19/18	31/19	23/15	0.579
Duration (year)	9.05 ± 6.35	12.10 ± 7.55	-	0.066
Onset age (year)	17.59 ± 9.21	16.80 ± 8.73	-	0.837
Preoperative seizure frequency	3.61 ± 3.89	4.06 ± 3.39	-	0.337
Preoperative seizure duration (minutes)	1.37 ± 0.75	1.63 ± 0.92	-	0.186
Follow-up time (year)	4.73 ± 2.93	5.26 ± 2.20	-	0.187
Lesion side			-	0.857
Left	20 (54.1)	28 (56.0)		
Right	17 (45.9)	22 (44.0)		
Hippocampal atrophy			-	0.838
Yes	23 (62.2)	30 (60.0)		
No	14 (37.8)	20 (40.0)		
Ebrile seizures			-	0.499
Yes	8 (21.6)	14 (28.0)		
No	29 (78.4)	36 (72.0)		
Meningitis			-	0.559
Yes	1 (2.7)	4 (8.0)		
No	36 (97.3)	46 (92.0)		
History of brain trauma			-	0.907
Yes	4 (10.8)	7 (14.0)		
No	33 (89.2)	43 (86.0)		

^*^p value < 0.05. SF, seizure-free; NSF, non-seizure-free; HC, healthy controls.

### 3.2 Metabolic network connectivity analysis

**Figure 1:**
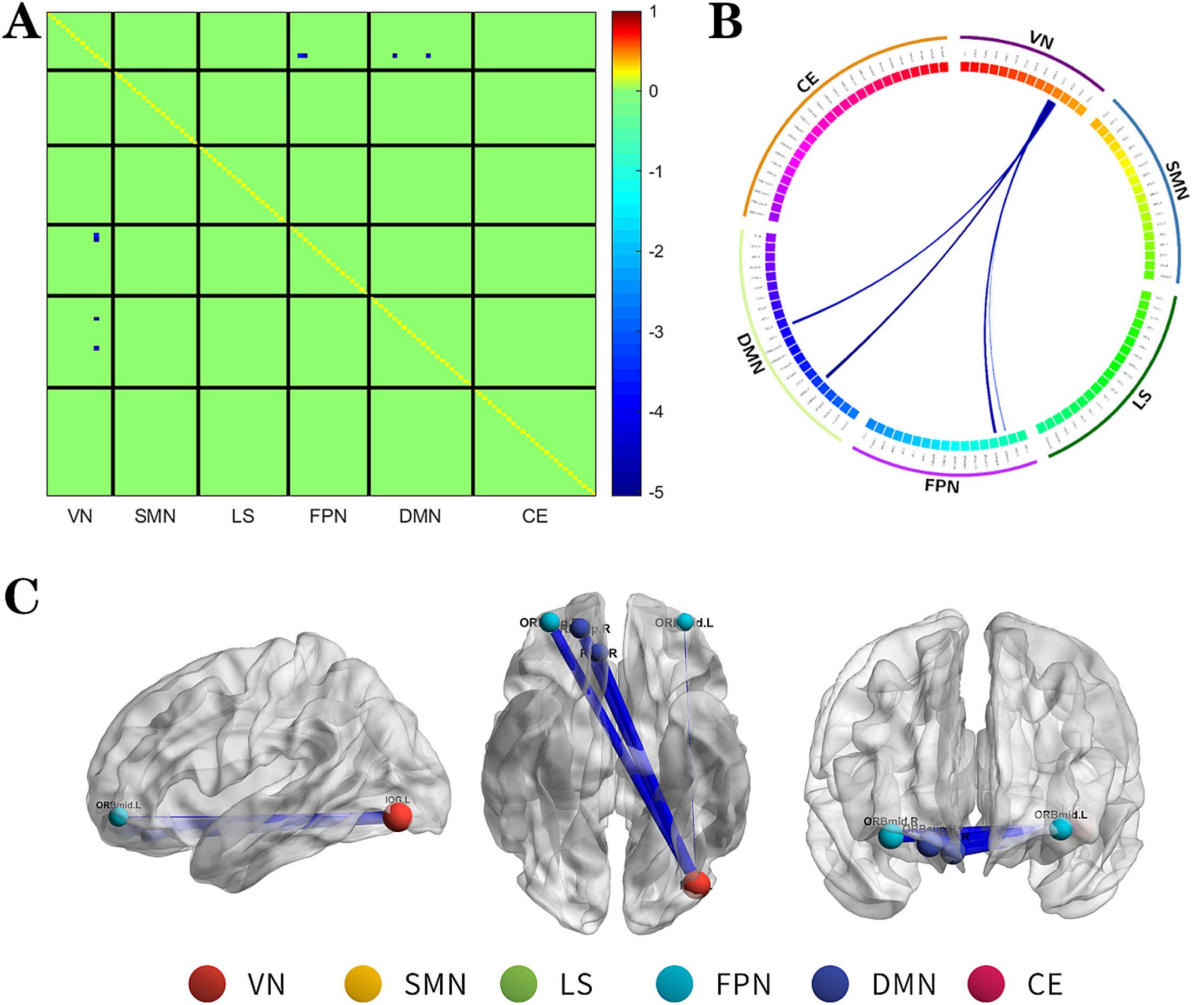
The changes metabolic correlation network between the NSF group and SF group. **(A–C)** represent the connectivity matrix maps, Circos and connectivity graph of the metabolic connectivity comparison between the NSF group and SF group. Compared to the SF group, the NSF group shows that the metabolic connections between the DMN, FPN, and VN were decreased. The blue nodes indicates the reduced metabolic connectivity, and the horizontal axis shows the brain network to which these abnormal metabolic connections belong **(A)**. The outer circle is divided into six brain networks, and the inner circle represents 116 brain regions **(B)**. The blue line indicates decreased connectivity, and the line width represents the differences in significant T values (*p* < 0.017, FDR corrected) **(B,C)**. DMN, default mode network; LS, limbic system; FPN, frontoparietal network; VN, visual network; SMN, sensorimotor network.

**Figure 2:**
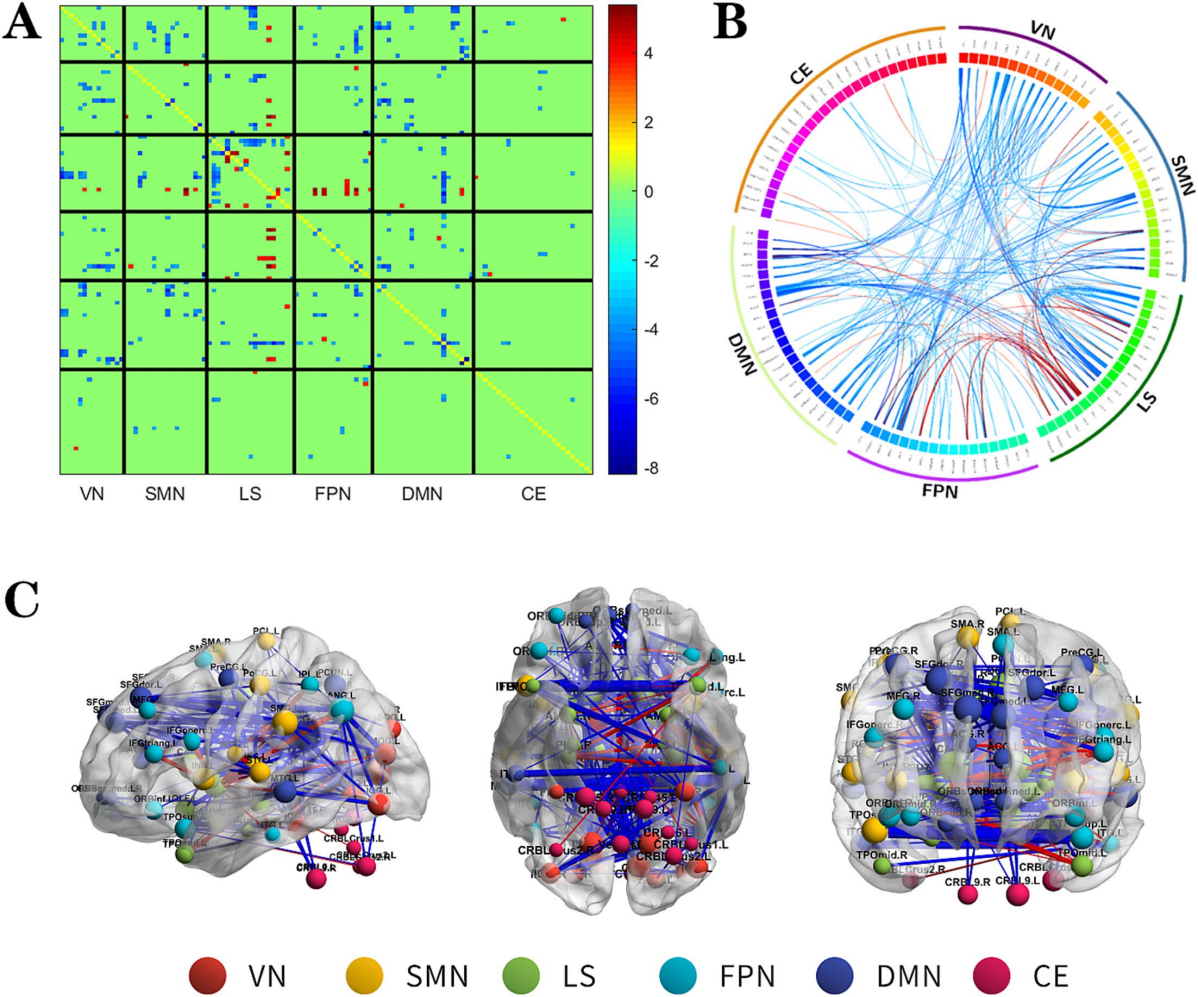
The changes metabolic correlation network between the NSF group and HC group. **(A–C)** represent the connectivity matrix maps, Circos and connectivity graph of the metabolic connectivity comparison between the NSF group and HC group. Compared to the HC group, the NSF group shows that abnormal metabolic connections were found in the DMN, FPN, VN, SMN, LS, and CE. The blue nodes represent reduced metabolic connectivity, and the red nodes indicate increased metabolic connectivity **(A)**. The red line indicates increased connectivity and the blue line indicates decreased connectivity, and the line width represents the differences in significant T values (*p* < 0.017, FDR corrected) **(B,C)**. DMN, default mode network; LS, limbic system; FPN, frontoparietal network; VN, visual network; SMN, sensorimotor network.

**Figure 3:**
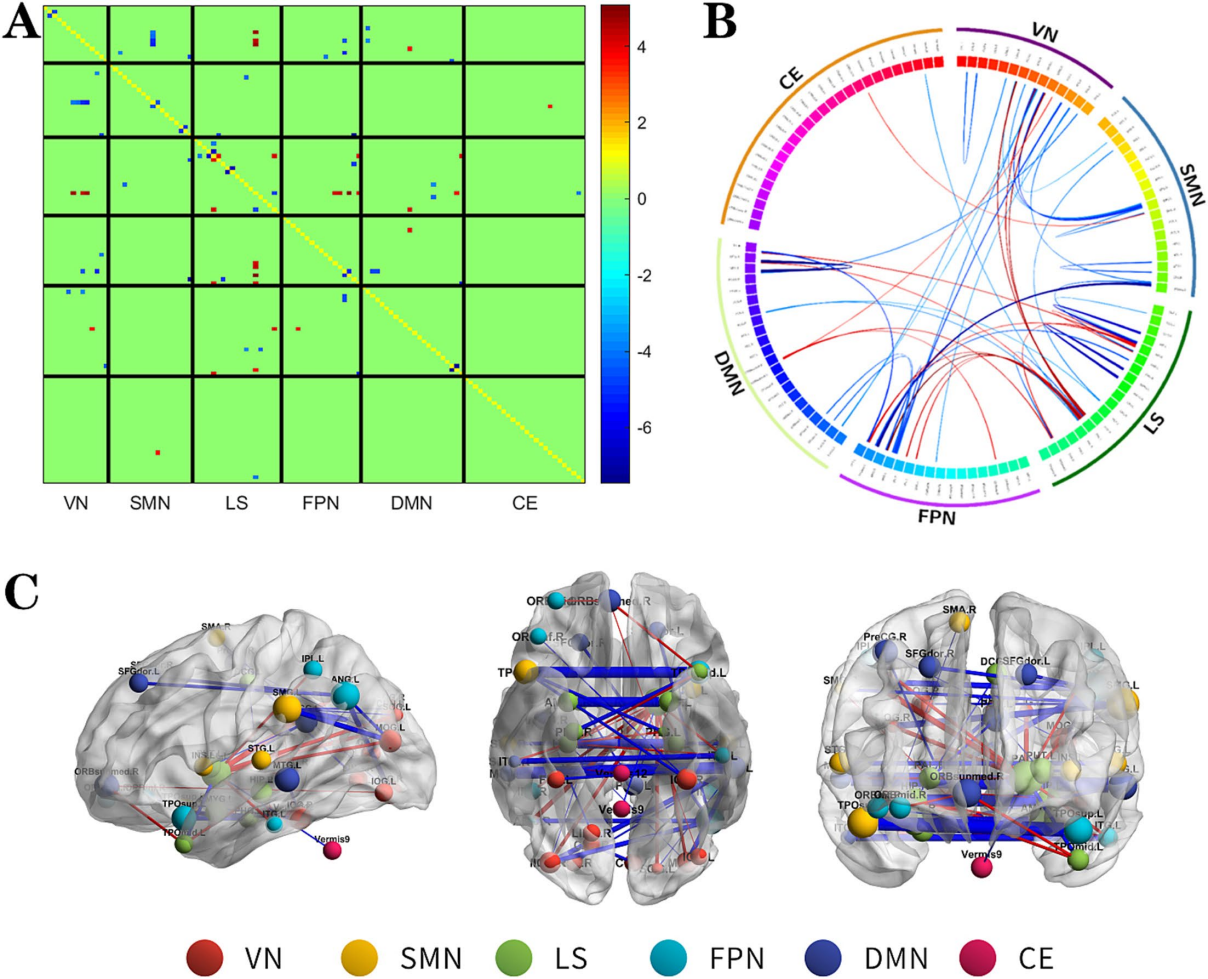
The changes metabolic correlation network between the SF group and HC group. **(A–C)** represent the connectivity matrix maps, Circos and connectivity graph of the metabolic connectivity comparison between the NSF group and SF group. DMN, default mode network; LS, limbic system; FPN, frontoparietal network; VN, visual network; SMN, sensorimotor network.

Inter-group comparison of the brain metabolic network connectivity revealed significant alterations in the DR-MTLE patients. The key affected regions belonged to the limbic system (LS), default mode network (DMN), frontoparietal network (FPN), sensorimotor network (SMN), visual network (VN), and cerebellum (CE) (see Figures 1–3; Supplementary Tables S1–S3 for detailed findings).

The NSF group demonstrated a marked decrease in metabolic connectivity compared to the SF group, particularly among the DMN, FPN, and VN (Figure 1, FDR correction *p* < 0.017).

Relative to the HC group, the NSF group exhibited widespread reductions in both intra-and inter-network metabolic connectivity, with notable decreases in the DMN, VN, SMN, and LS. Among them, the brain networks with weakened metabolic connections were located mainly within LS, between DMN and SMN, VN and between SMN and VN in the NSF group, which most significantly between DMN and VN. Conversely, regions of increased metabolic connectivity were mainly localized within the LS, between the LS and FPN, and between the LS and SMN, with the most prominent increase found between the LS and FPN (Figure 2, FDR correction *p* < 0.017).

Compared to the HC group, the SF group also showed significant alterations in metabolic connectivity, with both decreased and increased connectivity across the DMN, FPN, VN, SMN, LS, and cerebellum (CE). Among them, the metabolic network connections located mainly between DMN, SMN and VN, within LS and among other brain networks were weakened in the SF group, which most significantly between SMN and VN; and the metabolic network connections located mainly between LS and FPN, DMN, VN were enhanced in the SF group, which most significantly between LS and FPN (Figure 3, FDR correction *p* < 0.017).

These findings suggest pronounced disruptions in the metabolic connectivity of patients with DR-MTLE, particularly in those with suboptimal surgical outcomes.

## 4 Discussion

In this study, the employment of ES as a methodology to construct individual metabolic brain networks using preoperative ^18^F-FDG PET data represents a significant advancement in understanding the metabolic underpinnings of DR-MTLE. Our findings illuminate the intricate alterations in metabolic connectivity associated with different surgical outcomes, offering novel insights into the neural mechanisms underlying postoperative seizure control.

Our analysis revealed marked decreases in metabolic connectivity in DR-MTLE patients, with the NSF group exhibiting more pronounced disruptions compared to the SF group. These alterations predominantly affected the LS, DMN, FPN, SMN, VN, and CE. The differential impact on these networks underscores the complexity of DR-MTLE as a disorder that extends beyond the focal epileptogenic zone, affecting broader brain networks that govern critical cognitive and perceptual functions.

The diminished connectivity within and among the DMN, FPN, and VN in the NSF group suggests a disrupted interplay between higher-order cognitive networks and lower-level perceptual networks. This disruption likely contributes to the inefficiency in information processing and integration, which may be a factor in the persistence of postoperative seizures (31). In this study, compared with the HC group, the brain networks with weakened metabolic connectivity in the NSF and SF groups all involved the DMN, and in the NSF group, the weakened connectivity was mainly concentrated on the connection of the DMN with the VN and SMN. Changes in abnormal metabolic connectivity were also observed within the DMN, as well as between the DMN and other brain networks in both groups. Decreased metabolic connectivity between the DMN and VN was observed in the NSF group compared with the SF group. The DMN, known for its activation during rest and deactivation during task-oriented activities, plays an important role in the MTLE network and is pivotal for maintaining intrinsic brain activity as well as facilitating higher-level cognitive functions. The impaired coordination between the DMN and other resting state networks (RSNs) in TLE patients (32, 33) may reflect alterations in long-term neural networks resulting from recurrent seizures and influencing surgical outcomes. It also explains why many TLE patients are susceptible to cognitive dysfunction.

Furthermore, the significant alterations observed within the LS highlight its critical role in TLE pathophysiology. The LS (34, 35), encompassing structures like the amygdala, hippocampus, and thalamus, is central to the initiation and propagation of epileptic activity in MTLE (36, 37). The abnormal patterns of metabolic connectivity observed in this study suggest a functional impairment within the LS, possibly contributing to clinical manifestations such as behavioral, memory, learning, and emotional changes in DR-MTLE patients.

In addition, we detected that connections in the VN-SMN were weaker in DR-MTLE patients than in HC. The SMN and VN are mainly involved in processing physical stimuli of external objects, as well as feeling internal perception and movement processes. A study proposed (38) that motor and visual connections were integrated into a multimodal integration network that links perception, action, and cognition in the human functional connectome. It may explain the poorer visuospatial memory in DR-MTLE patients.

In summary, our study provides compelling evidence that recurrent seizures in DR-MTLE patients resulted in significant alterations in the metabolic connectivity of brain networks. These findings enhance our understanding of the mechanisms underlying poor postoperative seizure control and highlight the potential of the analysis of metabolic brain networks on individual levels as a tool for preoperative assessments and predicting surgical outcomes in DR-MTLE.

### 4.1 Limitations and future directions

The significant age differences between the patient and control groups in this study presented a potential confounding factor, despite our efforts to account for age in the statistical analysis. Future research should aim to match participants by age or further investigate the impact of age on these findings. Additionally, longitudinal studies are needed to track the evolution of post-surgery changes in metabolic connectivity, as well as to explore their relationship with long-term seizure outcomes and quality of life in DR-MTLE patients. For instance, the data in this study only included follow-ups of one year or more, with varying durations among patients, which may impact the prognostic outcomes of the follow-ups and consequently lead to bias in the grouping of patients, thus affecting the experimental results. Therefore, future studies could consider establishing standardized follow-up dates (1, 3, and 5 years post-surgery) to mitigate this influence.

## Data Availability

The datasets presented in this study can be found in online repositories. The names of the repository/repositories and accession number(s) can be found in the article/Supplementary material.
