# Supplementary material for: Individual metabolic brain network abnormalities associated with drug-resistant mTLE vary in surgical outcomes
Source: Front Neurol. 2024 Dec 18;15:1444787. doi: 10.3389/fneur.2024.1444787 (PMC11688404; doi:10.3389/fneur.2024.1444787)
Supplement: Supplementary file 1 [file Table_1.DOCX]

Table 1: Differences in metabolic connections between NSF and SF groups

| AAL label | Region 1  （abbreviation） | Network 1 | Region 2  （abbreviation） | | Network 2 |
| --- | --- | --- | --- | --- | --- |
| 6_53 | ORBsup.R | DMN | IOG.L | VN | |
| 9_53 | ORBmid.L | FPN | IOG.L | VN | |
| 10_53 | ORBmid.R | FPN | IOG.L | VN | |
| 28_53 | REC.R | DMN | IOG.L | VN | |

Abbreviations: DMN:default mode network; FPN:frontoparietal network; VN:visual network

Tabel 2.1： Regions of decreased strength of metabolic connectivity in the NSF group compared with the HC group

| AAL label | Region 1  （abbreviation） | Network 1 | Region 2  （abbreviation） | Network 2 |
| --- | --- | --- | --- | --- |
| 2_3 | PreCG.R | FPN | SFGdor.L | DMN |
| 1_47 | PreCG.L | FPN | LING.L | VN |
| 1_48 | PreCG.L | DMN | LING.R | VN |
| 1_58 | PreCG.L | FPN | PoCG.R | SMN |
| 1_79 | PreCG.L | DMN | HES.L | SMN |
| 2_47 | PreCG.R | LS | LING.L | VN |
| 2_48 | PreCG.R | DMN | LING.R | VN |
| 2_61 | PreCG.R | LS | IPL.L | FPN |
| 2_63 | PreCG.R | LS | SMG.L | SMN |
| 2_64 | PreCG.R | LS | SMG.R | SMN |
| 2_65 | PreCG.R | LS | ANG.L | FPN |
| 2_68 | PreCG.R | VN | PCUN.R | DMN |
| 3_33 | SFGdor.L | VN | DCG.L | LS |
| 3_48 | SFGdor.L | VN | LING.R | VN |
| 3_57 | SFGdor.L | VN | PoCG.L | SMN |
| 3_63 | SFGdor.L | FPN | SMG.L | SMN |
| 3_65 | SFGdor.L | FPN | ANG.L | FPN |
| 4_7 | SFGdor.R | FPN | MFG.L | FPN |
| 4_11 | SFGdor.R | FPN | IFGoperc.L | FPN |
| 4_47 | SFGdor.R | SMN | LING.L | VN |
| 4_48 | SFGdor.R | SMN | LING.R | VN |
| 4_51 | SFGdor.R | SMN | MOG.L | VN |
| 4_63 | SFGdor.R | LS | SMG.L | SMN |
| 4_64 | SFGdor.R | LS | SMG.R | SMN |
| 4_65 | SFGdor.R | LS | ANG.L | SMN |
| 4_66 | SFGdor.R | LS | ANG.R | FPN |
| 4_79 | SFGdor.R | LS | HES.L | SMN |
| 6_55 | ORBsup.R | LS | FFG.L | VN |
| 7_35 | MFG.L | LS | PCG.L | DMN |
| 8_35 | MFG.R | LS | PCG.L | DMN |
| 8_48 | MFG.R | FPN | LING.R | VN |
| 10_35 | ORBmid.R | LS | PCG.L | DMN |
| 10_112 | ORBmid.R | LS | Vermis6 | CE |
| 11_12 | IFGoperc.L | FPN | IFGoperc.R | FPN |
| 11_24 | IFGoperc.L | FPN | SFGmed.R | DMN |
| 11_50 | IFGoperc.L | FPN | SOG.R | VN |
| 11_52 | IFGoperc.L | FPN | MOG.R | VN |
| 12_24 | IFGoperc.R | FPN | SFGmed.R | DMN |
| 12_43 | IFGoperc.R | FPN | CAL.L | VN |
| 13_24 | IFGtriang.L | FPN | SFGmed.R | DMN |
| 13_75 | IFGtriang.L | FPN | PAL.L | LS |
| 13_76 | IFGtriang.L | FPN | PAL.R | LS |
| 13_16 | IFGtriang.L | FPN | ORBinf.R | FPN |
| 15_16 | ORBinf.L | FPN | ORBinf.R | FPN |
| 15_35 | ORBinf.L | FPN | PCG.L | DMN |
| 16_55 | ORBinf.R | FPN | FFG.L | VN |
| 16_85 | ORBinf.R | FPN | MTG.L | DMN |
| 18_35 | ROL.R | DMN | PCG.L | DMN |
| 19_53 | SMA.L | FPN | IOG.L | VN |
| 19_105 | SMA.L | FPN | CRBL9.L | CE |
| 19_106 | SMA.L | FPN | CRBL9.R | CE |
| 20_52 | SMA.R | DMN | MOG.R | VN |
| 20_53 | SMA.R | DMN | IOG.L | VN |
| 20_105 | SMA.R | DMN | CRBL9.L | CE |
| 20_106 | SMA.R | DMN | CRBL9.R | CE |
| 21_29 | OLF.L | LS | INS.L | SMN |
| 21_30 | OLF.L | LS | INS.R | SMN |
| 21_23 | OLF.L | LS | SFGmed.L | DMN |
| 21_35 | OLF.L | LS | PCG.L | DMN |
| 23_35 | SFGmed.L | DMN | PCG.L | DMN |
| 23_43 | SFGmed.L | DMN | CAL.L | VN |
| 23_44 | SFGmed.L | DMN | CAL.R | VN |
| 23_63 | SFGmed.L | DMN | SMG.L | SMN |
| 23_81 | SFGmed.L | DMN | STG.L | SMN |
| 23_82 | SFGmed.L | DMN | STG.R | SMN |
| 24_35 | SFGmed.R | DMN | PCG.L | DMN |
| 24_43 | SFGmed.R | DMN | CAL.L | VN |
| 24_44 | SFGmed.R | DMN | CAL.R | VN |
| 24_48 | SFGmed.R | DMN | LING.R | VN |
| 24_63 | SFGmed.R | DMN | SMG.L | SMN |
| 24_65 | SFGmed.R | DMN | ANG.L | FPN |
| 24_79 | SFGmed.R | DMN | HES.L | SMN |
| 24_81 | SFGmed.R | DMN | STG.L | SMN |
| 25_84 | ORBsupmed.L | DMN | TPOsup.R | SMN |
| 26_37 | ORBsupmed.R | DMN | HIP.L | LS |
| 29_32 | INS.L | SMN | ACG.R | DMN |
| 29_38 | INS.L | SMN | HIP.R | LS |
| 29_72 | INS.L | SMN | CAU.R | LS |
| 29_73 | INS.L | SMN | PUT.L | LS |
| 29_74 | INS.L | SMN | PUT.R | LS |
| 30_71 | INS.R | SMN | CAU.L | LS |
| 30_72 | INS.R | SMN | CAU.R | LS |
| 31_72 | ACG.L | DMN | CAU.R | LS |
| 32_35 | ACG.R | DMN | PCG.L | DMN |
| 33_38 | DCG.L | LS | HIP.R | LS |
| 33_41 | DCG.L | LS | AMYG.L | LS |
| 33_42 | DCG.L | LS | AMYG.R | LS |
| 33_71 | DCG.L | LS | CAU.L | LS |
| 33_72 | DCG.L | LS | CAU.R | LS |
| 33_73 | DCG.L | LS | PUT.L | LS |
| 33_74 | DCG.L | LS | PUT.R | LS |
| 33_76 | DCG.L | LS | PAL.R | LS |
| 33_78 | DCG.L | LS | THA.R | LS |
| 33_98 | DCG.L | LS | CRBL45.R | CE |
| 34_38 | DCG.R | LS | HIP.R | LS |
| 34_42 | DCG.R | LS | AMYG.R | LS |
| 34_71 | DCG.R | LS | CAU.L | LS |
| 34_72 | DCG.R | LS | CAU.R | LS |
| 34_73 | DCG.R | LS | PUT.L | LS |
| 34_76 | DCG.R | LS | PAL.R | LS |
| 34_78 | DCG.R | LS | THA.R | LS |
| 34_98 | DCG.R | LS | CRBL45.R | CE |
| 35_36 | PCG.L | DMN | PCG.R | DMN |
| 35_37 | PCG.L | DMN | HIP.L | LS |
| 35_38 | PCG.L | DMN | HIP.R | LS |
| 35_67 | PCG.L | DMN | PCUN.R | DMN |
| 35_68 | PCG.L | DMN | MTG.L | DMN |
| 35_71 | PCG.L | DMN | CAU.L | LS |
| 35_72 | PCG.L | DMN | CAU.R | LS |
| 35_73 | PCG.L | DMN | PUT.L | LS |
| 35_74 | PCG.L | DMN | PUT.R | LS |
| 35_75 | PCG.L | DMN | PAL.L | LS |
| 35_76 | PCG.L | DMN | PAL.R | LS |
| 35_77 | PCG.L | DMN | THA.L | LS |
| 35_78 | PCG.L | DMN | THA.R | LS |
| 35_97 | PCG.L | DMN | CRBL45.L | CE |
| 35_98 | PCG.L | DMN | CRBL45.R | CE |
| 36_72 | PCG.R | DMN | CAU.R | LS |
| 37_38 | HIP.L | LS | HIP.R | LS |
| 38_40 | HIP.R | LS | PHG.R | LS |
| 38_43 | HIP.R | LS | CAL.L | VN |
| 39_84 | PHG.L | LS | TPOsup.R | SMN |
| 40_83 | PHG.R | LS | TPOsup.L | FPN |
| 41_42 | AMYG.L | LS | AMYG.R | LS |
| 42_43 | AMYG.R | LS | CAL.L | VN |
| 43_51 | CAL.L | VN | MOG.L | VN |
| 43_67 | CAL.L | VN | PCUN.R | DMN |
| 43_68 | CAL.L | VN | MTG.L | DMN |
| 43_71 | CAL.L | VN | CAU.L | LS |
| 43_72 | CAL.L | VN | CAU.R | LS |
| 43_82 | CAL.L | VN | STG.R | SMN |
| 44_67 | CAL.R | VN | PCUN.R | DMN |
| 44_68 | CAL.R | VN | MTG.L | DMN |
| 44_71 | CAL.R | VN | CAU.L | LS |
| 44_72 | CAL.R | VN | CAU.R | LS |
| 45_71 | CUN.L | VN | CAU.L | LS |
| 45_72 | CUN.L | VN | CAU.R | LS |
| 47_50 | LING.L | VN | SOG.R | VN |
| 47_52 | LING.L | VN | MOG.R | VN |
| 47_57 | LING.L | VN | PoCG.L | SMN |
| 47_67 | LING.L | VN | PCUN.R | DMN |
| 47_68 | LING.L | VN | MTG.L | DMN |
| 47_69 | LING.L | VN | PCL.L | SMN |
| 47_71 | LING.L | VN | CAU.L | LS |
| 47_72 | LING.L | VN | CAU.R | LS |
| 47_98 | LING.L | VN | CRBL45.R | CE |
| 47_99 | LING.L | VN | CRBL6.L | CE |
| 48_50 | LING.R | VN | SOG.R | VN |
| 48_52 | LING.R | VN | MOG.R | VN |
| 48_57 | LING.R | VN | PoCG.L | SMN |
| 48_58 | LING.R | VN | PoCG.R | SMN |
| 48_64 | LING.R | VN | SMG.R | SMN |
| 48_66 | LING.R | VN | ANG.R | FPN |
| 48_67 | LING.R | VN | PCUN.R | DMN |
| 48_71 | LING.R | VN | CAU.L | LS |
| 48_72 | LING.R | VN | CAU.R | LS |
| 49_58 | SOG.L | VN | PoCG.R | SMN |
| 49_65 | SOG.L | VN | ANG.L | SMN |
| 49_93 | SOG.L | VN | CRBLCrus2.L | CE |
| 50_63 | SOG.R | VN | SMG.L | SMN |
| 50_65 | SOG.R | VN | ANG.L | SMN |
| 50_79 | SOG.R | VN | HES.L | SMN |
| 51_52 | MOG.L | VN | MOG.R | VN |
| 51_58 | MOG.L | VN | PoCG.R | SMN |
| 51_63 | MOG.L | VN | SMG.L | SMN |
| 51_65 | MOG.L | VN | ANG.L | SMN |
| 51_85 | MOG.L | VN | MTG.L | DMN |
| 52_63 | MOG.R | VN | SMG.L | SMN |
| 52_65 | MOG.R | VN | ANG.L | SMN |
| 53_63 | IOG.L | VN | SMG.L | SMN |
| 53_65 | IOG.L | VN | ANG.L | SMN |
| 53_66 | IOG.L | VN | ANG.R | FPN |
| 53_85 | IOG.L | VN | MTG.L | DMN |
| 53_86 | IOG.L | VN | MTG.R | DMN |
| 54_55 | IOG.R | VN | FFG.L | VN |
| 54_63 | IOG.R | VN | SMG.R | SMN |
| 54_65 | IOG.R | VN | ANG.L | SMN |
| 54_85 | IOG.R | VN | MTG.L | DMN |
| 55_84 | FFG.L | VN | TPOsup.R | SMN |
| 55_86 | FFG.L | VN | MTG.R | DMN |
| 56_85 | FFG.R | VN | MTG.L | DMN |
| 57_79 | PoCG.L | SMN | HES.L | SMN |
| 58_61 | PoCG.R | SMN | IPL.L | FPN |
| 58_65 | PoCG.R | SMN | ANG.L | SMN |
| 58_105 | PoCG.R | SMN | CRBL9.L | CE |
| 62_65 | SMG.L | SMN | ANG.L | SMN |
| 63_64 | SMG.R | SMN | SMG.R | SMN |
| 63_66 | SMG.R | SMN | ANG.R | FPN |
| 64_65 | SMG.R | SMN | ANG.L | SMN |
| 65_66 | ANG.L | SMN | ANG.R | FPN |
| 65_93 | ANG.L | SMN | CRBLCrus2.L | CE |
| 69_105 | PCL.L | SMN | CRBL9.L | CE |
| 71_81 | CAU.L | LS | STG.L | SMN |
| 71_112 | CAU.L | LS | Vermis6 | CE |
| 72_81 | CAU.R | LS | STG.L | SMN |
| 72_82 | CAU.R | LS | STG.R | SMN |
| 75_87 | PAL.L | LS | TPOmid.L | LS |
| 81_82 | STG.L | SMN | STG.R | SMN |
| 83_84 | TPOsup.L | FPN | TPOsup.R | SMN |
| 83_90 | TPOsup.L | FPN | ITG.R | DMN |
| 83_93 | TPOsup.L | FPN | CRBLCrus2.L | CE |
| 84_85 | TPOsup.R | SMN | MTG.L | DMN |
| 84_94 | TPOsup.R | SMN | CRBLCrus2.R | CE |
| 85_86 | MTG.L | DMN | MTG.R | DMN |
| 88_89 | TPOmid.R | LS | ITG.L | FPN |
| 89_90 | ITG.L | FPN | ITG.R | DMN |
| 98_112 | CRBL45.R | CE | Vermis6 | CE |

Abbreviations: DMN:default mode network; LS:limbic system; FPN:frontoparietal network; VN:visual network; SMN:sensorimotor network.

Table 2.2: Regions of increased strength of metabolic connectivity in the NSF group compared with the HC group

| AAL label | Region 1  （abbreviation） | Network 1 | Region 2  （abbreviation） | Network 2 |
| --- | --- | --- | --- | --- |
| 11_75 | IFGoperc.L | FPN | PAL.L | LS |
| 11_76 | IFGoperc.L | FPN | PAL.R | LS |
| 17_79 | ROL.L | DMN | HES.L | SMN |
| 13_32 | IFGtriang.L | FPN | ACG.R | DMN |
| 18_65 | ROL.R | DMN | ANG.L | SMN |
| 21_87 | OLF.L | LS | TPOmid.L | LS |
| 22_87 | OLF.R | DMN | TPOmid.L | LS |
| 38_39 | HIP.R | LS | PHG.L | LS |

| AAL label | | Region 1  （abbreviation） | Network 1 | Region 2  （abbreviation） | | Network 2 | |
| --- | --- | --- | --- | --- | --- | --- | --- |
| 38_87 | HIP.R | | LS | | TPOmid.L | | LS |
| 38_89 | HIP.R | | LS | | ITG.L | | FPN |
| 40_42 | HIP.R | | LS | | AMYG.R | | LS |
| 42_87 | PHG.R | | VN | | TPOmid.L | | LS |
| 46_110 | CAL.L | | VN | | Vermis3 | | CE |
| 48_75 | CUN.R | | VN | | PAL.L | | LS |
| 51_75 | LING.R | | VN | | PAL.L | | LS |
| 61_73 | MOG.L | | FPN | | PUT.L | | LS |
| 61_74 | IPL.L | | FPN | | PUT.R | | LS |
| 61_75 | IPL.L | | FPN | | PAL.L | | LS |
| 61_76 | IPL.L | | FPN | | PAL.R | | LS |
| 63_75 | IPL.L | | SMN | | PAL.L | | LS |
| 65_75 | SMG.R | | SMN | | PAL.L | | LS |
| 65_76 | ANG.L | | SMN | | PAL.R | | LS |
| 72_91 | ANG.L | | LS | | CRBLCrus1.L | | CE |
| 75_79 | CAU.R | | LS | | HES.L | | SMN |
| 75_81 | PAL.L | | LS | | STG.L | | SMN |
| 75_85 | PAL.L | | LS | | MTG.L | | DMN |
| 75_89 | PAL.L | | LS | | ITG.L | | FPN |
| 76_77 | PAL.L | | LS | | THA.L | | LS |
| 76_79 | PAL.R | | LS | | HES.L | | SMN |
| 83_94 | PAL.R | | FPN | | CRBLCrus2.R | | CE |
| 76_85 | TPOsup.L | | LS | | MTG.L | | DMN |

Table 3.1: Regions of increased strength of metabolic connectivity in the SF group compared with the HC group

| AAL label | Region 1  （abbreviation） | Network 1 | Region 2  （abbreviation） | Network 2 |
| --- | --- | --- | --- | --- |
| 10_26 | ORBmid.R | FPN | ORBsupmed.R | DMN |
| 26_53 | ORBsupmed.R | DMN | IOG.L | VN |
| 26_87 | ORBsupmed.R | DMN | TPOmid.L | LS |
| 38_39 | HIP.R | LS | PHG.L | LS |
| 38_87 | HIP.R | LS | TPOmid.L | LS |
| 38_89 | HIP.R | LS | ITG.L | FPN |
| 38_90 | HIP.R | LS | ITG.R | DMN |
| 49_75 | SOG.L | VN | PAL.L | LS |
| 51_75 | MOG.L | VN | PAL.L | LS |
| 52_75 | MOG.R | VN | PAL.L | LS |
| 61_75 | IPL.L | FPN | PAL.L | LS |
| 62_75 | SMG.L | SMN | PAL.L | LS |
| 64_109 | SMG.R | SMN | Vermis12 | CE |
| 66_75 | ANG.R | FPN | PAL.L | LS |
| 75_86 | PAL.L | LS | MTG.R | DMN |
| 75_89 | PAL.L | LS | ITG.L | FPN |

Abbreviations:DMN:default mode network; LS:limbic system; FPN:frontoparietal network; VN:visual network; SMN:sensorimotor network.

Table 3.2: Regions of decreased strength of metabolic connectivity in the SF group compared with the HC group

| AAL label | Region 1  （abbreviation） | Network 1 | Region 2  （abbreviation） | | Network 2 |  |
| --- | --- | --- | --- | --- | --- | --- |
| 2_48 | PreCG.R | DMN | LING.R | VN | |  |
| 2_51 | PreCG.R | DMN | MOG.L | VN | |  |
| 3_65 | SFGdor.L | DMN | ANG.L | SMN | |  |
| 4_65 | SFGdor.R | DMN | ANG.L | SMN | |  |
| 16_55 | ORBinf.R | FPN | FFG.L | VN | |  |
| 20_54 | SMA.R | DMN | IOG.R | VN | |  |
| 29_73 | INS.L | SMN | PUT.L | LS | |  |
| 33_38 | DCG.L | LS | HIP.R | LS | |  |
| 35_73 | PCG.L | DMN | PUT.L | LS | |  |
| 35_76 | PCG.L | DMN | PAL.R | LS | |  |
| 37_58 | HIP.L | LS | PoCG.R | SMN | |  |
| 39_83 | PHG.L | LS | TPOsup.L | FPN | |  |
| 39_84 | PHG.L | LS | TPOsup.R | SMN | |  |
| 41_42 | AMYG.L | LS | AMYG.R | LS | |  |
| 44_45 | CAL.R | VN | CUN.L | VN | |  |
| 49_63 | SOG.L | VN | SMG.R | SMN | |  |
| 50_63 | SOG.R | VN | SMG.R | SMN | |  |
| 51_63 | MOG.L | VN | SMG.R | SMN | |  |
| 51_65 | MOG.L | VN | ANG.L | SMN | |  |
| 52_63 | MOG.R | VN | SMG.L | SMN | |  |
| 54_65 | IOG.R | VN | ANG.L | SMN | |  |
| 56_85 | FFG.R | VN | MTG.L | DMN | |  |
| 63_64 | SMG.R | SMN | SMG.R | SMN | | |
| 65_66 | ANG.L | SMN | ANG.R | FPN | | |
| AAL label | Region 1  （abbreviation） | Network 1 | Region 2  （abbreviation） | | Network 2 |  |
| 75_87 | PAL.L | LS | TPOmid.L | | LS |  |
| 75_115 | PAL.L | LS | Vermis9 | | CE |  |
| 81_82 | STG.L | SMN | STG.R | | SMN |  |
| 83_84 | TPOsup.L | FPN | TPOsup.R | | SMN |  |
| 85_86 | MTG.L | DMN | MTG.R | | DMN |  |

Abbreviations: DMN:default mode network; LS:limbic system; FPN:frontoparietal network; VN:visual network; SMN:sensorimotor network.
